# A 3 km spatially and temporally consistent European daily soil moisture reanalysis from 2000 to 2015

**DOI:** 10.1038/s41597-020-0450-6

**Published:** 2020-04-03

**Authors:** Bibi S. Naz, Stefan Kollet, Harrie-Jan Hendricks Franssen, Carsten Montzka, Wolfgang Kurtz

**Affiliations:** 1 Jülich Research Center GmbH, Institute of Bio- and Geosciences: Agrosphere (IBG 3), Jülich, 52425 Germany; 2 Centre for High-Performance Scientific Computing in Terrestrial Systems, Geoverbund ABC/J, Jülich, 52425 Germany; 3 Leibniz Supercomputing Centre, Boltzmannstr. 1, 85748 Garching, Germany

**Keywords:** Hydrology, Scientific data

## Abstract

High-resolution soil moisture (SM) information is essential to many regional applications in hydrological and climate sciences. Many global estimates of surface SM are provided by satellite sensors, but at coarse spatial resolutions (lower than 25 km), which are not suitable for regional hydrologic and agriculture applications. Here we present a 16 years (2000–2015) high-resolution spatially and temporally consistent surface soil moisture reanalysis (ESSMRA) dataset (3 km, daily) over Europe from a land surface data assimilation system. Coarse-resolution satellite derived soil moisture data were assimilated into the community land model (CLM3.5) using an ensemble Kalman filter scheme, producing a 3 km daily soil moisture reanalysis dataset. Validation against 112 *in-situ* soil moisture observations over Europe shows that ESSMRA captures the daily, inter-annual, intra-seasonal patterns well with RMSE varying from 0.04 to 0.06 m^3^m^−3^ and correlation values above 0.5 over 70% of stations. The dataset presented here provides long-term daily surface soil moisture at a high spatiotemporal resolution and will be beneficial for many hydrological applications over regional and continental scales.

## Abstract (summary)

**Table:** 

Measurement(s)	wetness of soil
Technology Type(s)	digital curation • computational modeling technique
Factor Type(s)	geographic location • day
Sample Characteristic - Environment	soil
Sample Characteristic - Location	Europe

Machine-accessible metadata file describing the reported data: 10.6084/m9.figshare.11993547

## Background & Summary

Soil moisture (SM) is characterized by complex dynamics across a wide range of spatial and temporal scales^1^ that can impact hydrological processes such as runoff, evaporation and transpiration from vegetation through changing soil moisture^2^. As a result, accurate characterization of spatial distribution and temporal variations of SM is critical for many regional–scale applications, including weather predictions, subsurface hydrology, flood forecasting, drought monitoring, agriculture and climate change impact studies^1–3^. However, SM remains a difficult variable to obtain over large scale with reasonable temporal and spatial resolution, because there are no high-resolution soil moisture observations available at the continental scale and observations of SM from measurements are very sparse. While remote sensing (RS) products give reliable estimates and cover large areas^4–6^, most long-term soil moisture data from spaceborne remote sensors have relatively low spatial resolution (in the order of 25 to 50 km) and they are spatially and temporally discontinuous^7,8^. An alternative source of high-resolution SM estimates is from land surface models (LSM). However, predictions are often poor due to inadequate model physics, poor parameter estimates and erroneous atmospheric forcings^9^. Soil moisture reanalysis products are therefore needed which can provide downscaled estimates of SM with complete spatiotemporal coverage by merging coarse-resolution SM observations with a high resolution LSM using data assimilation (DA) techniques^3,10–13^. These products overcome the shortcomings of sparse spatial and temporal distributions in observations and provide a better estimate of SM than obtained only by modeling or by satellite observations alone. Soil moisture reanalysis provides unique and consistent datasets for studying complex spatial patterns of SM from regional to global scales and temporal variability from daily to annual scales^1,14^. Moreover, the relationship to other essential climate variables, such as runoff or evapotranspiration, can be investigated in more detail. It can also be used as initial input for climate change analysis and numerical simulations and for cross-validation of SM outputs in modeling studies.

Several commonly used long-term soil moisture global datasets exist from land surface DA systems^15–17^, as listed in Table 1. The focus of these reanalysis systems has been on the assimilation of meteorological observations, except for the Global Land Evaporation Amsterdam Model (GLEAM, v3.2a)^18^ product which assimilate surface SM. The overall goal of these products is to provide estimates of atmospheric, land and oceanic climate variables. At the European regional scale, there have been few studies which provide soil moisture reanalysis through DA techniques by assimilating surface soil moisture information from satellite into land surface models^10,19–23^. Though these global and regional reanalysis products are an attractive data source, they have a relatively coarse resolution (typically at 25–50 km grid spacing) and may not provide locally representative information of soil moisture which is important for regional hydrologic and agriculture applications.

**Table 1:** Main characteristics of commonly used long-term soil moisture global datasets from reanalysis products.

Product	ERA5	MERRA2-land	GLEAM-3.2a	GLDAS-2.1	NCEP-CFSR
Time period	1979–present	1980–present	1980–2018	2000–2019	1979–present
Spatial resolution (km^2^)	~31	~50	25	25	50
Spatial coverage	Global	Global	Global	Global	Global
Temporal resolution	Hourly	Hourly	Daily	3 hourly	3 hourly
Assimilation scheme	4D-VAR	3D-VAR	Newtonian nudging	EnKF	3D-VAR

The land surface DA system CLM-PDAF consisting of the Community Land Model (CLM)^24^ and the Parallel Data Assimilation Framework (PDAF)^25,26^ was used to utilize the coarse resolution satellite soil moisture data to update the soil moisture estimates in the land surface model. The DA structure in CLM-PDAF allows to directly ingest remotely sensed observations of land surface conditions to produce accurate, spatially and temporally consistent fields of land surface states, with reduced uncertainties through an ensemble based DA method. Recently, the European Space Agency Climate Change Initiative (ESACCI)^27^ provides a homogeneous and the longest time series of SM data to date, covering the period 1979–2018, and has been widely used for various Earth system research^28–30^ and in DA studies^19,31,32^. We selected ESACCI SM data for DA because of its availability at longer timescales, which also makes it possible to construct a long-term high-resolution SM reanalysis at continental scale.

**Fig. 1:**
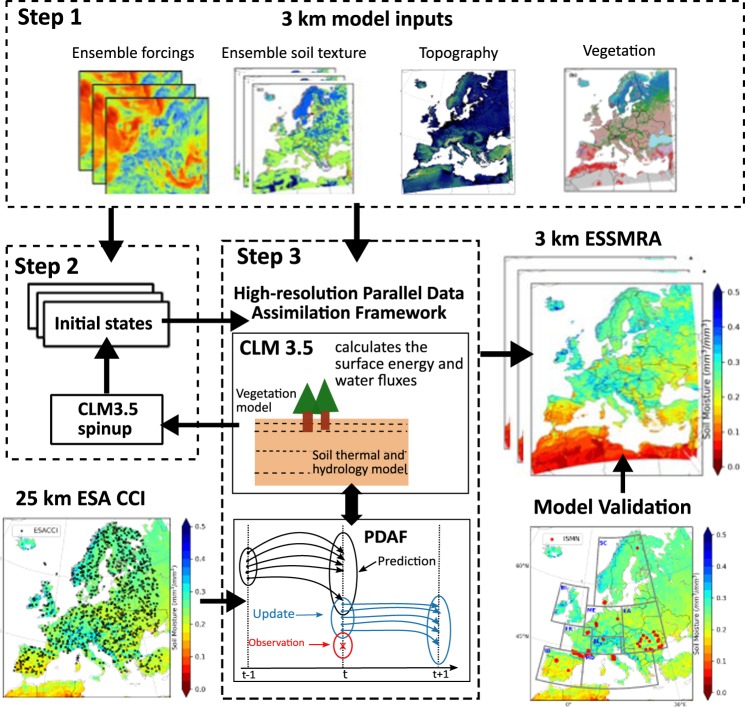
Schematic of CLM-PDAF workflow adopted to generate high resolution ESSMRA product. The first step was to prepare an ensemble of 3 km input data for the CLM model for the EU-CORDEX domain. Second, CLM was initialized for each ensemble member with the equilibrium initial state variables. In the third step, selected ESA CCI soil moisture observations (resolution 25 km) were assimilated into CLM using the Ensemble Kalman filter to generate the 3 km ESSMREA dataset.

Using CLM-PDAF, the daily SM data at 0.25° resolution from ESACCI were assimilated into CLM using an ensemble Kalman filter (EnKF) DA method^33,34^ producing the first 3 km European SSM reanalysis (called ESSMRA hereafter) dataset. Figure 1 shows the schematic flow of the CLM-PDAF setup to develop the ESSMRA dataset. The purpose of ESSMRA is to provide a long-term (2000–2015) spatially and temporally consistent data source with high spatiotemporal resolution (3 km, daily) and high quality for the research community to use in hydrological and climate applications as well as to study the spatial/temporal variability of SM over Europe. The relatively longer time scale and fine spatial resolution of this new European gridded ESSMRA dataset could provide a valuable data source for many hydrological applications over larger regimes and to regional and continental scale studies.

## Methods

The 3 km ESSMRA was generated using three main steps: (1) the regional land surface model setup over Europe, (2) implementation of a DA framework, and (3) validation of ESSMRA based on observations and other reanalysis products.

### Land surface model, parameters and data

The community land model (CLM), available through the National Centre of Atmospheric Research (NCAR) as part of the Community Climate System Model (CCSM) is used in this soil moisture reanalysis system. The coupled land surface data assimilation system (CLM-PDAF) uses the CLM version3.5 (CLM3.5) which offers significant improvements in estimating the components of the terrestrial water cycle compared to earlier versions (i.e. CLM2.0 and 3.0)^24^. Later versions of CLM (4.0 and 4.5) may be used in the future, however, a previous study showed that the differences between CLM3.5 and later versions of CLM (4.0 and 4.5) with respect to soil moisture variability remained small when compared to observations^35^. The CLM3.5 simulates the hydrological cycle over land by taking into account interception of water by plants, throughfall, infiltration, runoff, soil water and accumulation and melting of snow. In CLM3.5 the soil profile is divided into 10 soil layers (0–3.8 m). The input soil texture information (sand fraction, clay fraction) is available for the surface layer only. For simplicity, sand fraction and clay fraction information for 19 soil classes in the first layer were also used for the deeper layers. The movement of moisture between these layers is calculated using Richard’s equation. The bottom soil layer is also coupled with an unconfined aquifer to account for groundwater recharge and discharge processes^24^. The current setup of CLM3.5 does not consider different geological conditions of bedrock.

To account for land surface variability within a grid cell, a CLM grid cell consists of one or more columns to capture surface heterogeneity through land unit (e.g. glacier, wetland, lake, and vegetation). The vegetated fraction can be further divided into 17 different plant functional types (PFTs). The water and energy balance equations are solved for each land cover type and aggregated back to the grid cell level. CLM requires several static surface input parameters related to vegetation, soils and topography^36^. Table 2 lists the source for each input parameter used in this study. The land cover information for each PFT in our model setup was estimated based on the Moderate Resolution Imaging Spectro radiometer (MODIS) MCD12Q1 (version5) land cover product^37^. The 1 km Global Land Surface Satellite (GLASS) LAI (leaf area index) product^38^ was used to estimate 12 monthly LAI values for every 3 km grid cell which allow spatially distributed monthly LAI values for each PFT. Additionally, yearly model runs were performed where the LAI information was updated at the start of each year to account for annual variability in LAI. Additional properties such as the stem area index and the monthly heights of each PFT were calculated based on the global CLM3.5 surface dataset^24^. Soil texture data such as sand and clay percentages were determined for 19 soil classes derived from the Food and Agricultural Organization (FAO) database^39^ and soil characteristics dataset developed by Miller *et al*.^40^. Topography data were acquired from the 1 km Global Multi-resolution Terrain Elevation Data 2010 (GMTED2010)^41^.

**Table 2:** Model input datasets used to generate ESSMRA.

Dataset	Spatial resolution (km)	Temporal Resolution	Source	model input
COSMO-REA6	6	Hourly	ftp://opendata.dwd.de	Meteorological Inputs
MODIS (MCD12Q1)	0.5	Yearly		Land Cover
GLASS LAI	1	Weekly	http://glcf.umd.edu	LAI
FAO Soil	10	NA	http://www.fao.org	Soil Texture
GMTED2010	1	NA	https://www.usgs.gov/	DEM

The atmospheric forcing data such as solar radiation, temperature, pressure, near-surface wind speed, specific humidity and precipitation rate were prepared using the regional atmospheric reanalysis COSMO-REA6 dataset^42^ from the Hans Ertel Centre for Weather Research (HErZ)^43^. It is based on the numerical weather prediction COSMO (Consortium for Small Scale Modelling) model^44^ and spans the period 1995–2017 with hourly data of atmospheric variables at 0.055° (∼6 km) over Europe. The COSMO-REA6 was corrected through the assimilation of observational meteorological data using the existing nudging scheme in COSMO model with boundary conditions from ERA-Interim data. A more comprehensive description of the dataset is available in previous studies^42,45^.

The SM satellite observations from the combined ESACCI dataset at 0.25° resolution were used for the DA experiment, as described in the next section. In the past decade, a number of different satellite missions have been launched to provide SM retrievals with high temporal resolution over large regions. Examples are Soil Moisture and Ocean Salinity^46,47^ (SMOS; launched in 2009) and the Soil Moisture Active Passive (SMAP, launched in 2015) missions^48^. The complete list of operational remotely sensed surface SM products is also given in Babaeian *et al*.^8^. These recent data products are only available for the last decade and cannot be used to apply soil moisture information in a land surface reanalysis for extended time periods. The combined ESACCI product which combines both active and passive microwave sensors provides large spatiotemporal coverage and offers a good opportunity to improve LSM estimates with DA techniques. In this merged product, the absolute soil moisture values are rescaled to common climatology using soil moisture estimates from GLDAS-Noah model through CDF-matching method. The quality of the ESACCI SM product has been evaluated on a global scale by several studies^28^. By comparing the ESACCI SM dataset with *in-situ* measurements, a previous study found that the product was able to capture the annual cycle of SM and its short-term variability^29^. The quality of the product has been shown to increase with time due to the addition of new satellites and methods used to merge them^28^.

### Generation of ESSMRA dataset using CLM-PDAF

For the generation of the ESSMRA dataset, we used the CLM–PDAF framework, in which PDAF is coupled with CLM for soil moisture assimilation. The ESSMRA product was generated by performing three main steps as shown in Fig. 1. First, CLM3.5 was implemented for the EURO-CORDEX domain with a spatial resolution of 0.0275° (3 km), inscribed into the official EUR11 grid. The model was driven with COSMO-REA6 reanalysis dataset for the time period 2000–2015. To match the spatial resolution of CLM3.5 setup, the COSMO-REA6 dataset at 6 km resolution was re-gridded to 0.0275° (3 km) using the first-order conservative interpolation method^49^. In the second step, a 30 years spin-up run (simulating time period of 2000–2006 five times) of CLM forced by atmospheric fields was carried out to obtain equilibrium initial state variables which were used to initialize the model. In the third step, the model was run for 2000–2015 at hourly time step with the assimilation of ESACCI soil moisture data into the model once a day using the EnKF algorithm. The EnKF algorithm uses ensembles of model simulations to approximate the model state and parameter error covariance matrix in order to optimally merge model predictions with observations^3,50–52^. The PDAF is designed for high-performance computing infrastructures and can efficiently cope with the high computational burden of ensemble-based DA^25,26^. Because of this feature, it was possible to produce a pan-European longer-term and high spatial resolution land surface DA product. To generate ensembles of forecast states, we perturbed the precipitation and the soil parameters (sand and clay percentage) by applying log normally distributed multiplicative perturbations (with a mean of 1 and standard deviation of 0.15) to the precipitation field and random noise drawn from spatially uniform distribution (±10%) to the sand and clay content, respectively. In the present study, we only updated the soil moisture state variable and kept the soil texture constant for individual ensemble members throughout our simulations instead of joint state and parameter updates of soil moisture and soil texture in the DA approach as used in our previous study^31^. The ensemble size was set to 20 in our assimilation experiment using similar methodology as used in Naz *et al*.^31^. Our initial study found slightly improved SM estimates when ensemble size was increased from 12 to 20^31^. However, increasing ensemble size is quite challenging for such a large-scale high-resolution model because of the memory and storage requirements. In the DA approach, another challenge is the spatial mismatch between coarse-resolution satellite data and high-resolution hydrologic models. To address the resolution mismatch between the ESACCI data and model (i.e. 0.25° and 0.0275°, respectively), the ESACCI grid cell nearest to the model grid cell was identified and considered as an independent data point for DA. While this approach avoids the additional step of downscaling the satellite data to model resolution, it may smooth out the high-resolution features of the LSM. In future multiscale assimilation (i.e. to update various model grid cells covered by a satellite observation) of the ESACCI SM data into CLM could be explored^53^. Another limitation associated with our method is that due to the large number of grid cells (1544 × 1590) and required computational resources, it was not possible to assimilate all of the data available from the ESACCI satellite data into CLM. In the current framework, we randomly selected 1000 grid cells (5% of total grid cells over land). The SM observations at the selected grid cells were then assimilated into the CLM model. The non-assimilated data were used in the model validation step. This approach allowed us to evaluate the impact of DA at other locations where the data were not assimilated. However, it should be noted that this approach might negatively affect the SM estimates that are further apart from the assimilated grid cells because of the use of global EnKF in our approach. In the future, the local ensemble transform Kalman filter (LETKF) could be used to avoid this limitation. The strength and limitations associated with our methods are also discussed in detail in Naz *et al*.^31^.

**Fig. 2:**
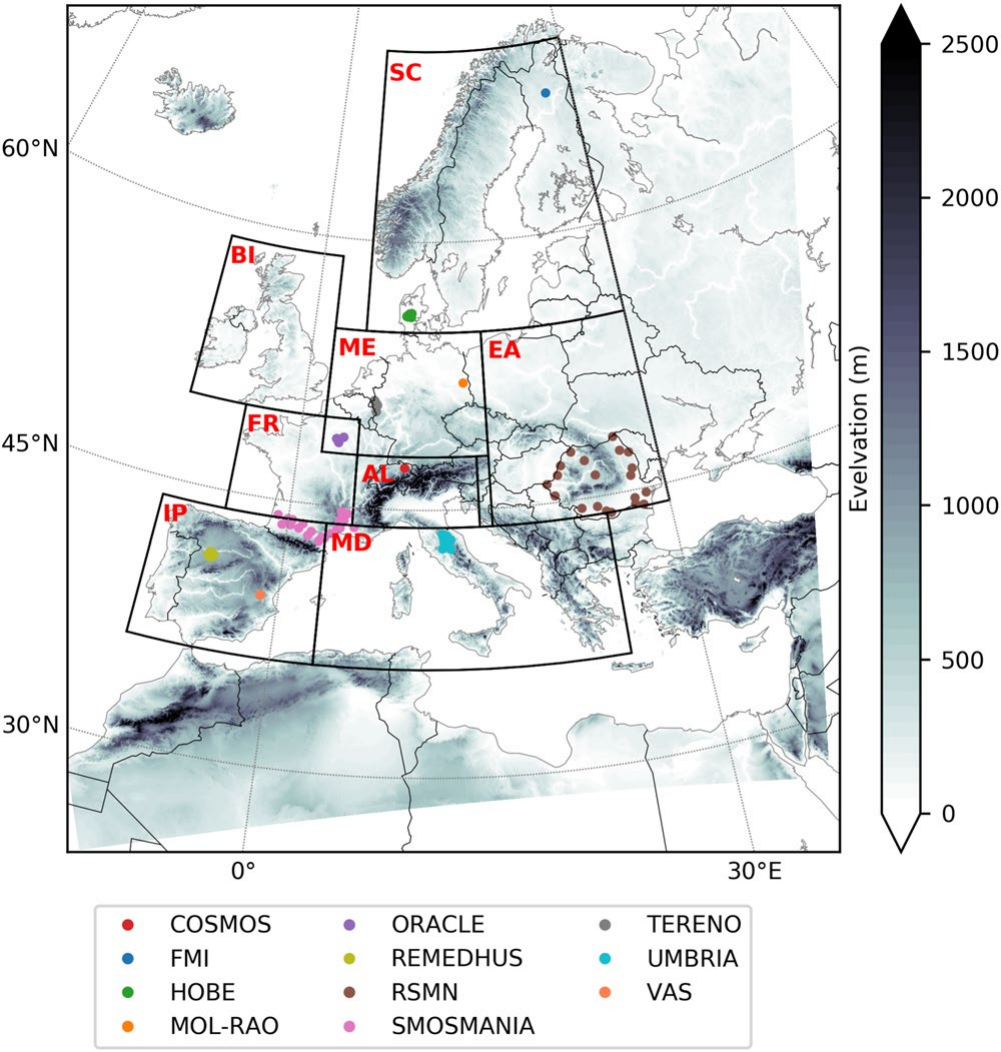
Map of EURO-CORDEX domain (1544 × 1592 grid cells) showing surface elevation, boundaries of PRUDENCE regions and locations of International Soil Moisture Network (ISMN) stations used for data validation. Black boxes correspond to PRUDENCE regions with abbreviated letters in red colour indicating names of the regions (FR: France, ME: Mid-Europe, SC: Scandinavia, EA: Eastern Europe, MD: Mediterranean, IP: Iberian Peninsula, BI: the British Isles, AL: Alpine region. The solid dots represent locations of the *in-situ* ISMN soil moisture stations.

Using the above setup, DA experiments were conducted using CLM-PDAF over Europe (Fig. 2). We selected 2000–2015 as our period of analysis because of the availability of most model input data in our experiment for this time period. This experiment allowed us to generate a 16-year high resolution ESSMRA dataset at daily time scale. A second experiment was also performed to evaluate the impact of DA using the same model setup, but without assimilating the ESACCI observations into the model. We referred to these experiments as “CLM-DA” (data assimilation) and “CLM-OL” (open loop simulation), respectively.

### Observation and reanalysis products

The *in-situ* soil moisture data from 11 networks across Europe were acquired from the International Soil Moisture Network (ISMN)^54^, which provides globally available soil moisture measurements. The surface soil moisture data from 112 stations for the top 5 to 10 cm surface layer were collected to evaluate the ESSMRA product in the top two CLM soil layers (about 3 cm). *In-situ* data were collected for 2000–2015, but their availability does not necessarily cover the whole period. For comparison with ESSMRA daily estimates, the measurements with hourly time scale were aggregated to daily time scale. If more stations are located within one 3 km grid cell, the average of those stations was used for comparison. The characteristics of the selected *in-situ* networks are presented in Table 3.

**Table 3:** Information of selected *in-situ* soil moisture stations used in the study.

Network	Country	# of Stations	Soil depth (m)	Start date	End date
COSOMO	Austria	1	0.0–0.1	2010-12-13	2015-12-31
FMI	Finland	1	0.0–0.05	2011-09-18	2015-12-31
HOBE	Denmark	23	0.0–0.05	2009-09-08	2015-01-01
MOL-RAO	Germany	1	0.0–0.08	2003-01-01	2014-01-01
ORACLE	France	3	0.0–0.05	2000-01-07	2013-09-09
REMEDHUS	Spain	24	0.0–0.05	2005-03-15	2015-12-31
RSMN	Romania	20	0.0–0.05	2014-04-09	2015-12-31
SMOSMANIA	France	21	0.0–0.05	2007-01-01	2015-12-31
TERENO	Germany	5	0.0–0.05	2009-12-31	2015-12-31
UMBRIA	Italy	13	0.0–0.05	2002-10-09	2015-12-31
VAS	Spain	2	0.0–0.05	2010-01-10	2012-01-01

The ESSMRA data were also compared with other global soil moisture reanalysis products from the European Centre for Medium-Range Weather Forecasts Reanalysis 5 (ERA5)^17^, Global Land Data Assimilation System (GLDAS)^15^ and Global Land Evaporation Amsterdam Model (GLEAMv3.2a)^18^ available at 0.25° resolution and hourly temporal resolution. The aim is to understand the spatiotemporal patterns of ESSMRA relative to other existing reanalysis products and how ESSMRA differs from other SM reanalysis products at regional scale. The SM from these reanalysis datasets have been widely used by many studies^18,55–62^. For instance, a comparison of GLDAS and ERA5 SM with *in-situ* data in Europe showed good agreement in terms of temporal dynamics of SM data^62^. Similarly, the SM estimates from GLEAM (v3) compared well with *in-situ* surface SM and was further improved than its previous versions with assimilation of surface soil moisture observations into the GLEAM^18^.

## Data Records

The ESSMRA dataset in NetCDF format is freely available for download from PANGAEA data repository^63^ as well as at the Jülich Supercomputing Centre data repository^64^. The dataset consists of ensemble mean of daily surface soil moisture for the period of 2000–2015 and is available at a monthly temporal frequency using the following naming convention as:

EU_ESSMRA_daily_ensmean_CLM–PDAF_3Km_v1.yyyymm.nc.

The netcdf files contain the variable “H2OSOIL” which is the volumetric soil moisture at the 0–3 cm layer [m^3^m^−3^]. For example the file, EU_ESSMRA_daily_ensmean_CLM–PDAF_3Km_v1.200101.nc contains the daily soil moisture values for the month of January 2001. Each file also contains the definition of the geographical coordinate system of the grid (latitudes, longitudes and rotated pole).

## Technical Validation

The newly developed ESSMRA dataset was validated at different spatiotemporal scales in four steps. First, the ESSMRA datset was validated using independent stations data. Second, the performance of ESSMRA was evaluated at regional scale with respect to ESACCI data to assess the impact of DA at other locations where the data were not assimilated. Third, the ability of ESSMRA to capture the monthly and yearly climatologies for different regions in Europe was evaluated against existing global reanalysis products (ERA5, GLDAS and GLEAM). Finally, ESSMRA was compared with ERA5, GLDAS and GLEAM reanalysis products to understand the spatial variability of SM at the European scale.

For evaluation against *in-situ* station measurements and other commonly used SM products we used the Pearson correlation coefficient (R), root mean square error (RMSE), unbiased root-mean-square error (ubRMSE) and a metric α proposed by Duveiller *et al*.^65^ which represents additive/multiplicative bias between two datasets. For α, 0 represents full bias and 1 indicates no bias. For this validation, we extracted the ESSMRA data to the nearest location of the station. However, if more stations are located within one 3 km grid cell, we used the average of those stations. For regional analysis, the results were presented for eight predefined analysis regions from the “Prediction of Regional scenarios and Uncertainties for Defining European Climate change risks and Effects” (PRUDENCE) project^66^ (FR: France, ME: Mid-Europe, SC: Scandinavia, EA: eastern Europe, MD: Mediterranean, IP: Iberian Peninsula, BI: British Isles, AL: Alpine region) as shown in Fig. 2. We referred to these regions as the “PRUDENCE” regions.

For ESSMRA validation, the average of simulated SM in the top two layers (i.e., at 0.007 and 0.03 m depth) of CLM was used. Because of high computational cost and storage requirement associated with implementing the continental scale 3 km integrated hydrologic and DA framework, currently, we only analysed the top 3 cm soil moisture, which is the limitation of this study. However, prior study^67^ showed no major differences for latent heat flux estimation using information content of surface SM or enhanced with soil moisture in deeper layers. With advancing capabilities in computing and storage, ESSMRA dataset can be extended to root zone data analysis using CLM-PDAF in the future.

### Validation using independent *in-situ* station observations

**Fig. 3:**
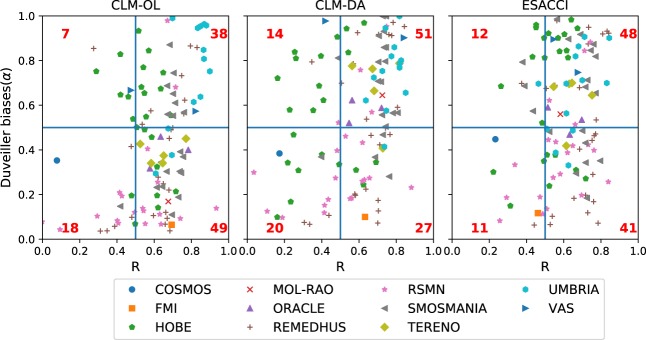
Scatter plot of R and α of surface SM from CLM-DA, CLM-OL and ESACCI against *in-situ* observations used in this study. Horizontal and vertical lines divide the graph into four quadrants based on thresholds of R and α. The total number of stations falling into each category is labelled in each quadrant of the graph. The stations are color-coded by soil moisture network.

The surface SM from CLM-DA and CLM-OL experiments were validated against *in-situ* measurements and also compared with the ESACCI satellite merged product (shown in Fig. 3). The surface SM observations were obtained from the ISMN database using all the data available over Europe between 2000 and 2015 (Table 3) and were used for the independent validation. The Pearson correlation coefficient (R) and α for each product were calculated at the *in-situ* station locations. These statistics were computed taking all station measurements into account for the period 2000–2015. Figure 3 shows the scatter plot of these scores against *in-situ* data for CLM-DA, CLM-OL and ESACCI.

Using a threshold of 0.5 for both R and α, stations fell into four categories, (1) stations with higher agreement (i.e. both R and α are greater than 0.5), (2) stations with higher R value but lower α values (i.e. there is higher difference in magnitude but temporal dynamics match well), (3) stations with higher values of α and low R values, and (4) stations with both low values of R and α. The thresholds for both R and α are based on the general rule of thumb that R > 0.5 represent moderate to high correlation. The performance of CLM-DA experiments in comparison to CLM-OL experiment and EASCCI data was evaluated in each of these four categories for bias and correlation. Based on these thresholds, Fig. 3 shows that SM from CLM-DA is in good agreement with observations over half of the stations (i.e. 51 out of 112 fell into category 1) for both R and α having values greater than 0.5 whereas CLM-OL and ESACCI shows higher agreement with observation for 38 and 48 stations, respectively. Overall CLM-DA performs well in matching the magnitude (i.e. α values > 0.5) over 58% of stations and higher correlation values for 70% of the stations, while ESACCI shows α values greater than 0.5 for 53% of the stations but exhibits higher correlation values (i.e. >0.5) for 80% of the stations. CLM-DA did a poor job for 17% of the stations where both magnitude and temporal dynamics are not matching well with observations (i.e. α and R values < 0.5). These locations were mostly for the *in-situ* stations of RSMN and HOBE networks, where ESSMRA SM is overestimated with respect to the stations data. This overestimation is also reported in other studies^68,69^ as well based on the comparison with satellite-based SM products.

Table 4 shows statistical scores for ESSMRA at all *in-situ* locations across all networks. On average, correlations ranged from 0.01 to 0.70 while α values ranged from 0.08 to 0.94. The average ubRMSE and RMSE values ranged from 0.04 to 0.06 m^3^m^−3^ and 0.05 to 0.12 m^3^m^−3^, respectively. A potential cause for lower correlation values of ESSMRA for some of the networks such as FMI, HOBE (located in Scandinavian region) and COSMOS (located in Alpine region) might be related to the limitations and uncertainties of ESACCI retrieval algorithm which is sensitive to vegetation, frozen soil and complex topography^27^. The SM in these areas is also influenced by soil freezing and thawing processes, dense forest, soil organic matter and the presence of numerous water bodies and bogs^69,70^. These processes are not well represented in the land surface models.

**Table 4:** Statistical scores for the comparison between ESSMRA and *in-situ* SM for all 112 stations available during 2000–2015.

Networks	R			alpha			RMSE			ubRMSE		
	min	average	max	min	average	max	min	average	max	min	average	max
REMEDHUS (24)	0.29	0.63	0.84	0.07	0.50	0.97	0.05	0.12	0.19	0.04	0.06	0.15
HOBE(23)	−0.01	0.31	0.69	0.11	0.56	0.93	0.05	0.09	0.23	0.04	0.06	0.12
SMOSMANIA (21)	0.40	0.70	0.79	0.28	0.67	1.00	0.04	0.09	0.17	0.04	0.06	0.09
RSMN(20)	0.01	0.49	0.88	0.09	0.34	0.95	0.06	0.12	0.18	0.04	0.06	0.08
UMBRIA(13)	0.63	0.75	0.82	0.40	0.74	1.00	0.04	0.09	0.16	0.04	0.07	0.12
TERENO(5)	0.57	0.67	0.79	0.42	0.68	0.77	0.06	0.07	0.09	0.05	0.06	0.07
ORACLE(3)	0.55	0.61	0.71	0.51	0.55	0.62	0.10	0.11	0.12	0.06	0.08	0.09
VAS(2)	0.41	0.63	0.84	0.90	0.94	0.98	0.05	0.06	0.07	0.04	0.05	0.07
COSMOS (1)	NA	0.09	NA	NA	0.35	NA	NA	0.12	NA	NA	0.08	NA
FMI (1)	NA	0.01	NA	NA	0.08	NA	NA	0.16	NA	NA	0.05	NA
MOL-RAO(1)	NA	0.70	NA	NA	0.64	NA	NA	0.08	NA	NA	0.05	NA

There are also some caveats regarding the use of *in-situ* observations for validation of model estimates. First, because of the differences in the spatial representativeness between different products, it is complicated to evaluate the coarser resolution product against point measurements i.e. the local measurement may not properly represent the large-scale average. For example at the point scale point, measurements cover ~1 dm^3^, while the model has a grid resolution of approximately 3 km. Second, CLM near-surface soil moisture variable represents an average over the top 5 cm of soil, whereas the *in-situ* measurements do not represent such a depth average, the surface soil moisture measurements instead represent conditions at a depth of about 5 cm. The spatial representativeness error and the vertical mismatch between the *in-situ* measurements and the modeled soil moisture variable will influence the skill metrics we computed for the validation.

**Fig. 4:**
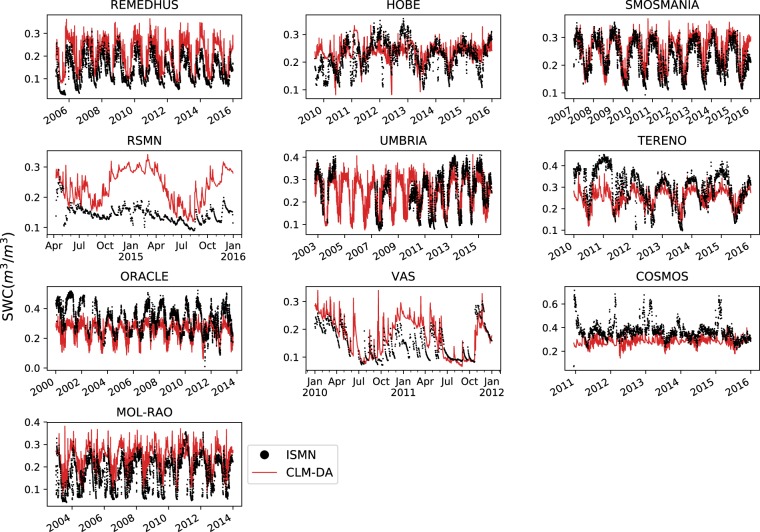
Comparison of daily time series of soil water content (m^3^/m^3^) from CLM-DA and *in-situ* observations from the ISMN networks. The average of the *in-situ* observations of all stations within the ISMN network was first calculated and then compared with the averaged soil moisture of all grids within the same ISMN network.

Despite these issues, ESSMRA product shows overall good agreement with *in-situ* observations at daily time scale as shown in Fig. 4. Because of the issues stated above with spatial representativeness, the average of the *in-situ* observations of all stations was compared with the averaged soil moisture of all grids within each ISMN listed network. This comparison shows that at daily scale the model is able to reproduce the daily variations in soil moisture fairly well, except for the RSMN network. The overestimation of ESSMRA SM data over RSMN is in line with findings by previous studies^68,69^.

### Regional validation using ESACCI SM data

The ESACCI SM data, which were excluded from the DA procedure, were used to evaluate the impact of assimilating soil water content (m^3^m^−3^; SWC) on model estimated SM at regional scale. We calculated the skill score (SS) of RMSE, ubRMSE, R and α for SWC using following equations:

1 $$S{S}_{RMSE}=1-\frac{D{A}_{RMSE}}{O{L}_{RMSE}}$$

2 $$S{S}_{ubRMSE}=1-\frac{D{A}_{ubRMSE}}{O{L}_{ubRMSE}}$$

3 $$S{S}_{R}={R}_{DA}-{R}_{OL}$$

4 $$S{S}_{\alpha }={\alpha }_{DA}-{\alpha }_{OL}$$

Positive SS values in Eq. (1) to Eq. (4) indicate improvement as a result of DA relative to the open loop, while SS < 0 indicates a degradation in assimilation results.

**Fig. 5:**
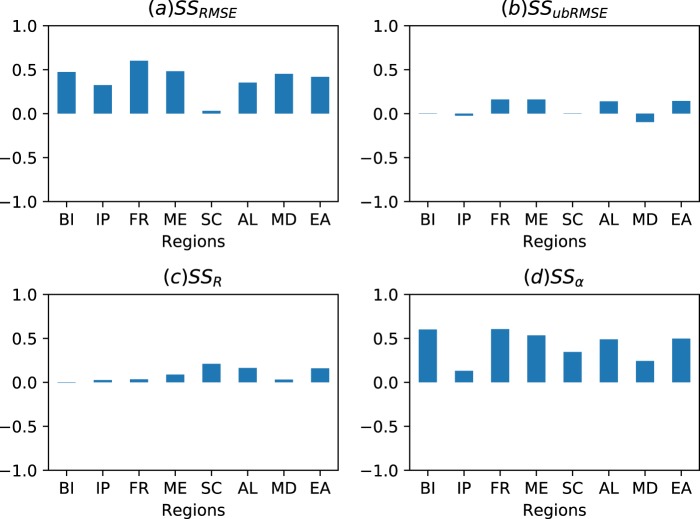
Performance evaluation of CLM-OL and CLM-DA against ESACCI SM. The skill score of (**a**) SS_RMSE_, (**b**) SS_ubRMSE_, (**c**) SS_R_ and (**d**) SS_α_ were calculated using spatially averaged SWC over each PRUDENCE region for years 2000–2015 from CLM-OL and CLM-DA simulations.

The impact of data assimilation on CLM-DA model performance is illustrated in Fig. 5 on the basis of skill scores for RMSE, ubRMSE, R and α for spatially averaged SWC over the PRUDENCE regions. For CLM-DA, assimilation of ESACCI observation shows positive SS_ubRMSE_ (i.e. reduced RMSE against CLM-OL) over all PRUDENCE regions with significant improvement over FR region and least improvement over SC region (Fig. 5a). However, assimilation of ESACCI had little impact on the model performance to capture the observed temporal variations in SWC as indicated by small positive values of SS_ubRMSE_ (ranges from 0.1 to 0.2) over regions FR, ME, AL and EA and negative values over BI, IP and MD regions (i.e. −0.02, −0.05 and −0.08, respectively; Fig. 5b). Figure 5c shows that assimilating ESACCI overall give little improvement in terms of correlation (SS_R_ > 0.02) with slightly degraded correlation over the BI region (−0.01). However, most regions show significant improvements in terms of reducing biases in SWC as indicated by positive SS_α_ values (>0.12) (Fig. 5d). The smaller improvements in R are likely due to the fact that temporal dynamics of CLM estimated SM are captured well in the CLM-OL, in which case there is little benefit from assimilation.

### Regional validation using reanalysis products

**Fig. 6:**
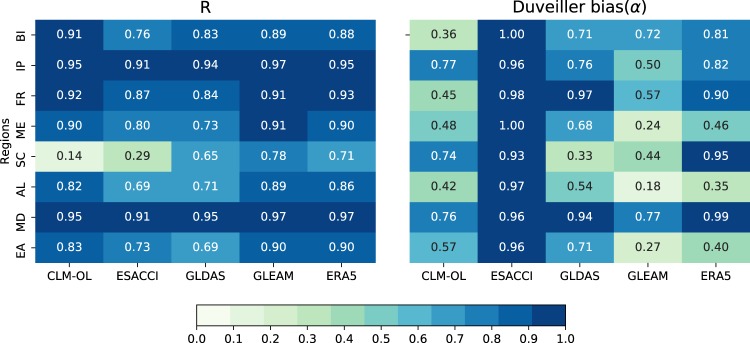
Performance evaluation of CLM-DA (ESSMRA) against GLDAS, GLEAM, ERA5, ESACCI and CLM-OL over PRUDENCE regions. Skill scores of (**a**) R and (**b**) Duveiller bias(α) were calculated using spatially averaged soil water content for years 2000–2015 over each PRUDENCE regions.

To further explore the quality of ESSMRA, we evaluated the skill of ESSMRA over PRUDENCE regions in comparison with commonly used SM reanalysis products as shown in Fig. 6. For this analysis, R and α were computed using spatially-averaged soil moisture over PRUDENCE regions between ESSMRA (CLM-DA) and other products (GLDAS, GLEAM and ERA5, ESACCI). This analysis shows that overall ESSMRA has higher correlation (i.e. R > 0.7) with other reanalysis products, which indicates higher agreement of ESMRA with other products in terms of timing and relative magnitude in time series. For the bias results, overall ESSMRA has higher agreement with ERA5 (α > 0.8), followed by GLDAS (α > 0.71), while it has lower agreement with GLEAM (α < 0.5) over most regions. Higher agreement with the ESACCI (α > 0.90) than the CLM-OL, again, shows the positive impact of DA through correcting of errors in the timing and magnitude of the soil moisture.

In addition, ESSMRA’s ability to capture the monthly and yearly climatologies over PRUDENCE regions is also evaluated for the period 2000–2015 against existing reanalysis products as shown in Supplementry Figs. S1 and S2. These results suggest that ESSMRA follows the seasonal variations fairly well, indicating that the timing and magnitude of SM at monthly and annual scales is reasonably accurate. As shown in Supplementry Fig. S1, however, in the dryer regions such as IP and MD, the soil moisture estimates by ESSMRA are lower than the other products particularly in summer. This might be due to the fact that satellite soil moisture tends to underestimate the true soil moisutue content in dry conditions due to systematic retrieval errors^71^ which may also affect the accuracy of assimilated SM estimation. On the other hand, the dry bias could be related to the different spatial scales and soil layers depths between the products. For example, in GLDAS the near-surface soil moisture variable represents an average over the top 10 cm of soil, whereas ERA5 and GLEAM estimate surface soil moisture over the 5 cm of soil depth. Moreover, small differences between SM estimates from these datasets might also be related to missmatch between input datasets, model parameterizations and assumptions used by different LSMs for generation of these products. Nevertheless, above results suggest overall a good agreement at daily, monthly and annual scale with SM renalysis products, which increases confidence in ESSMRA SM estimates.

### Soil moisture variability

To assess the ability of ESSMRA to capture short term soil moisture variability in comparison to other reanalysis products, seasonal and annual standardized anomalies (SMA) were calculated as follows:

5 $$SMA=\frac{S{M}_{t}-\overline{SM}}{{SM}_{\sigma }}$$

where *SM*_*t*_ is the average soil moisture value for a current year, $\overline{SM}$ is long-term average and ${SM}_{\sigma }$ is the standard deviation, which are both calculated for the same period of 2000–2015.

**Fig. 7:**
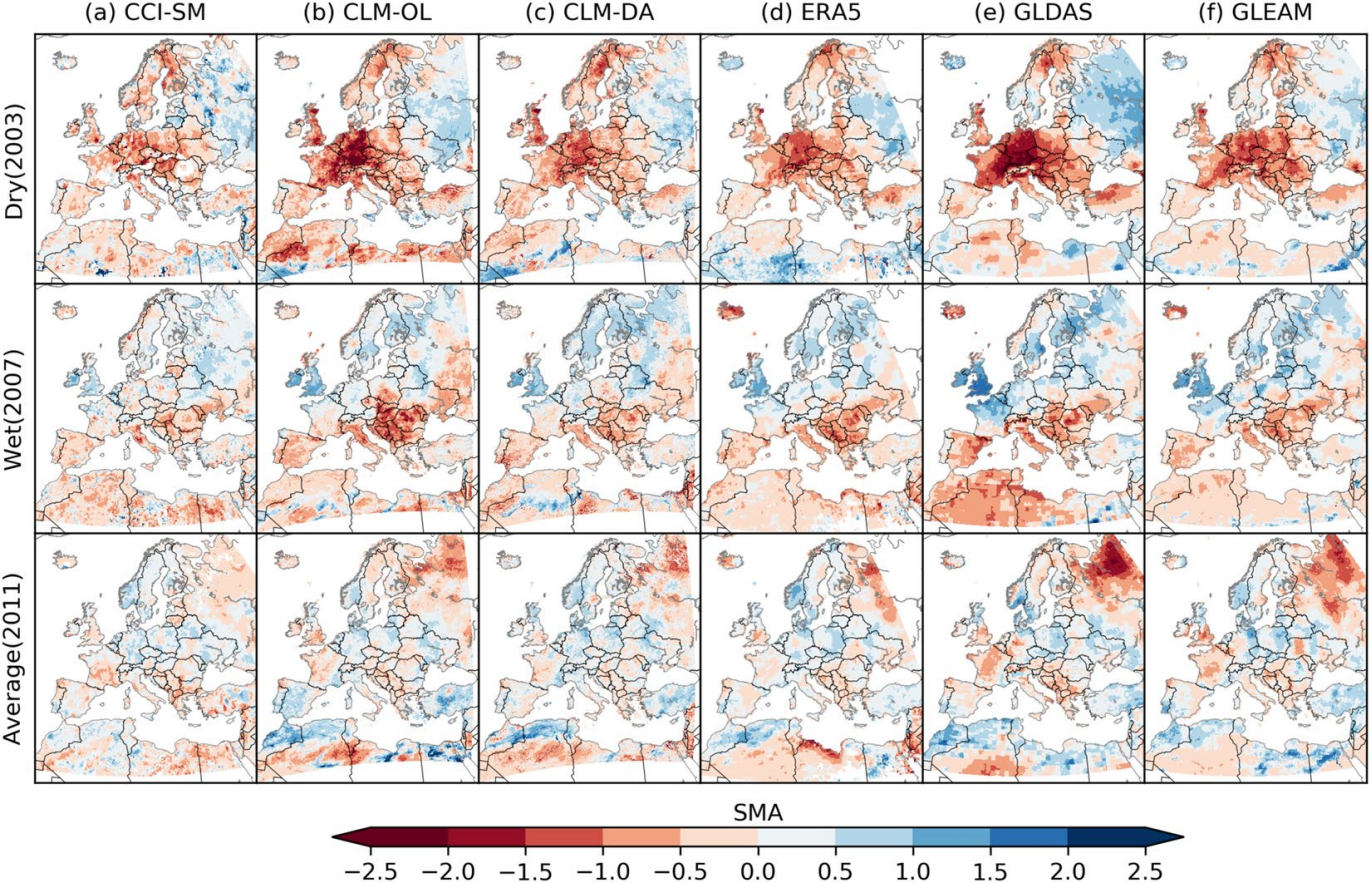
Spatial distribution of the standardized summer (JJA) soil moisture anomaly for dry, wet and average years. The soil moisture anomalies were calculated for year 2003 (dry year), 2007 (wet year) and 2011 (average year) and compared across existing data and reanalysis products of (**a**) satellite (ESACCI), (**b**) CLM-OL (**c**) CLM-DA, (**d**) ERA5, (**e**) GLDAS and (**f**) GLEAM.

Figure 7 illustrates the spatial distribution of SSM anomalies from the ESACCI satellite merged product, GLDAS, GLEAM, ERA5 and ESSMRA developed in this study along with CLM-OL. Summer anomalies of SM for a dry year (2003), wet year (2007) and average year (2011) were calculated using average SM values over June, July and August (JJA) relative to the mean JJA SM for the 2000–2015 period. The dry, wet and average years were selected by comparing yearly precipitation amounts to the long term average precipitation of 2000–2015 over Europe. The spatial distribution shows similar patterns of positive and negative SM anomalies over Europe across all datasets for dry, wet and average years. For the dry year 2003 (a record heat wave over Europe), CLM-DA shows a similar area extent of negative anomalies as the ESACCI dataset and ERA5, whereas CLM-OL, GLDAS and GLEAM exhibit much stronger negative anomalies over central Europe. The SM anomaly from CLM-DA for the wet and average years (2007, 2011) shows a good match with ESACCI and other reanalysis datasets except GLDAS which shows much stronger wet and dry anomalies than others.

**Fig. 8:**
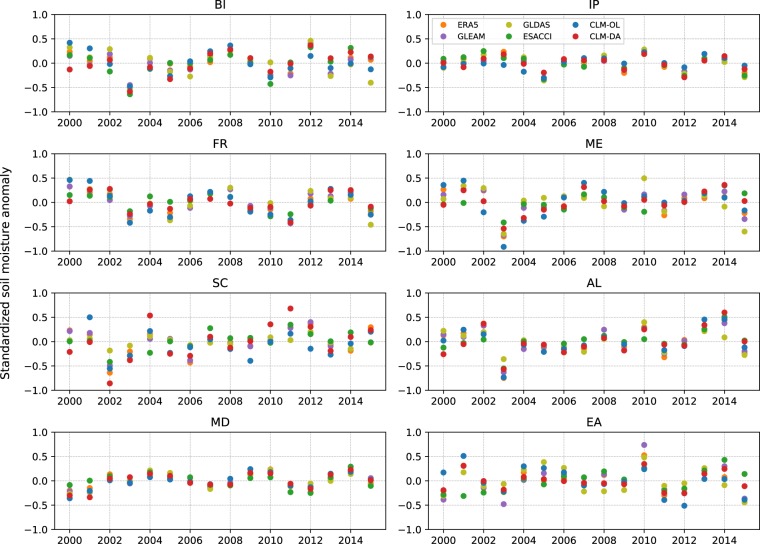
The standardized annual soil moisture anomalies from ESACCI, CLM-OL, CLM-DA, ERA5, GLDAS and GLEAM over the PRUDENCE regions. The standardized annual soil moisture anomalies were calculated using spatially averaged soil water content for years 2000–2015 over each PRUDENCE region.

At the annual scale, the time series of soil moisture anomalies over the PRUDENCE regions for 2000–2015 (Fig. 8) shows that SM anomalies from CLM-DA could capture the temporal SM variability and agrees well with SM anomalies from ESACCI, GLDAS, GLEAM and ERA5 across all regions. However, the agreement between different products was higher for the average years than for the dry and wet years.

## Usage Notes

The soil moisture dataset at high spatiotemporal resolution could be used for many practical applications. For example, it can be used as an initial input data for climate change analysis and for numerical weather prediction models to improve the model forecast in terms of location and amount of extreme precipitation events. Because of the scarcity of the *in-situ* soil moisture observations over large areas, this dataset can also be used for validation of SM outputs in modelling studies. This dataset will also be useful to understand the development and persistence of extreme weather events such as droughts, floods and heatwaves.

However, the ESSMRA dataset may still include some uncertainties. For example, uncertainties in ESSMRA may exist in regions where satellite soil moisture retrievals are sparse due to topography, standing water, dense vegetation, frozen soil and/or snow-covered areas. Apart from data gaps, ESACCI is a merged product and may contain inconsistencies because of differences in sensor characteristics and soil moisture retrieval algorithms^29^. These inconsistencies may also induce uncertainties in the ESSMRA data, particularly in some regions such as over Northern Europe or in Alpine regions and need to be improved in the future.

## Supplementary information

Supplementary information

## Data Availability

The CLM-PDAF setup is available through the Terrestrial System Modelling Platform (TSMP). TSMP is provided through a git repository available at the model’s website (https://www.terrsysmp.org/). The users are required to register to the git repository to get access to the code, pre- and post-processing tools and documentations for installing the code with examples setups. TSMP is released without the component models. For the coupled CLM-PDAF configuration, the code for PDAF library is available through website (pdaf.awi.de) which also provide links to the documentation and the source code. The CLM (version 3.5), as used in this study, is available as an open source model through the official CLM website (http://www.cgd.ucar.edu/tss/clm/distribution/clm3.5/index.html) which offers all links to documentation, source code, and input data for the stand-alone version release of CLM.
