# Supplementary material for: A 3 km spatially and temporally consistent European daily soil moisture reanalysis from 2000 to 2015
Source: Sci Data. 2020 Apr 3;7:111. doi: 10.1038/s41597-020-0450-6 (PMC7125156; doi:10.1038/s41597-020-0450-6)
Supplement: Supplementary file 1 — Supplementary information [file 41597_2020_450_MOESM1_ESM.pdf]

## List of Supplementary Figures

**Figure S1:** Comparison of monthly time series of soil water content ($m^3/m^3$) from ESSMRA (CLM-DA) with SM estimates from GLDAS, GLEAM, ERA5, ESACCI and CLM-OL over PRUDENCE regions for the period of 2000–2015. [2]

**Figure S2:** Comparison of long-term annual monthly average (2000-2015) of soil water content ($m^3/m^3$) from ESSMRA (CLM-DA) with SM estimates from GLDAS, GLEAM, ERA5, ESACCI and CLM-OL over the PRUDENCE regions. [3]

## Supplementary figures

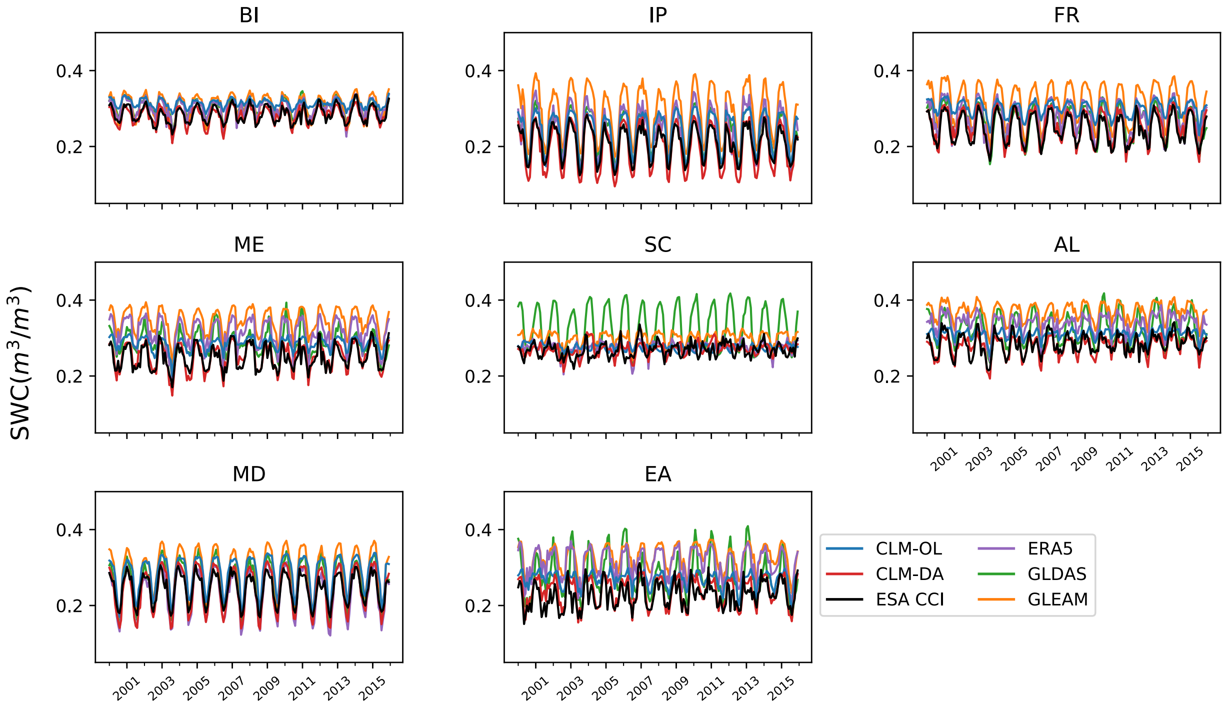


Figure S1: Comparison of monthly time series of soil water content ($m^3/m^3$) from ESSMRA (CLM-DA) with SM estimates from GLDAS, GLEAM, ERA5, ESACCI and CLM-OL over PRUDENCE regions for the period of 2000–2015.

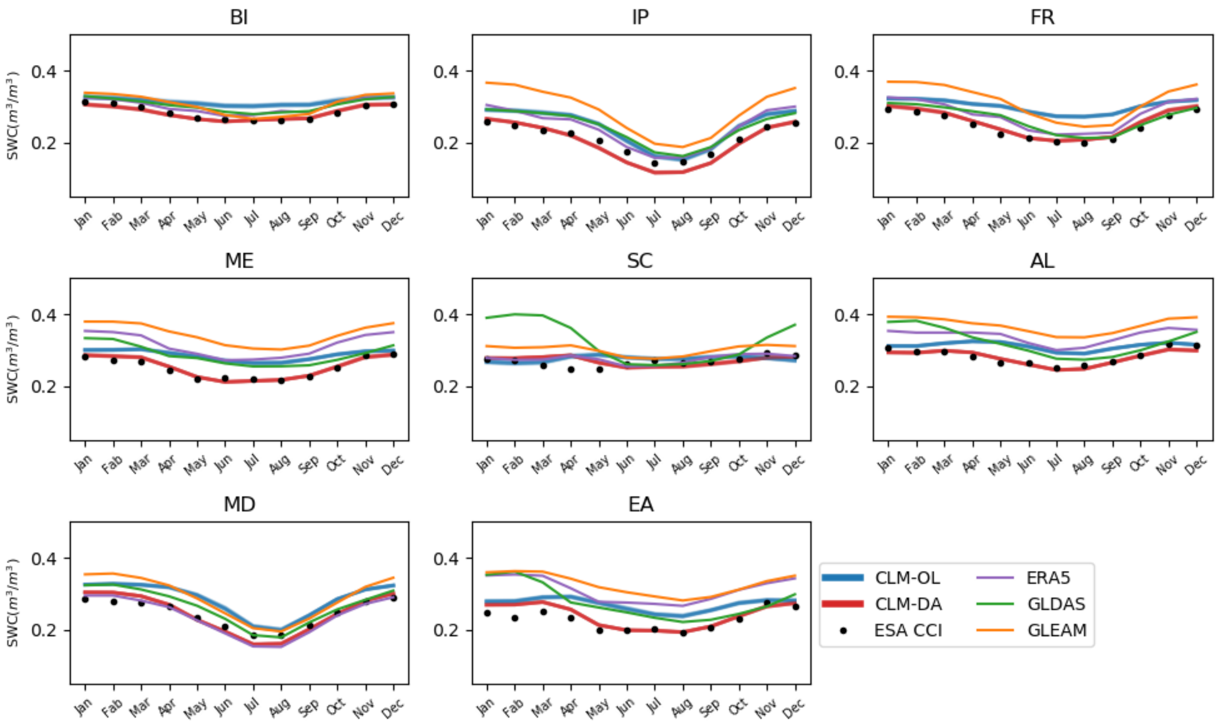


Figure S2: Comparison of long-term annual monthly average (2000–2015) of soil water content ($m^3/m^3$) from ESSMRA (CLM-DA) with SM estimates from GLDAS, GLEAM, ERA5, ESACCI and CLM-OL over the PRUDENCE regions.
